# Relationship Between Receiving Social Support and the Health of Grandparent Caregivers

**DOI:** 10.1093/geroni/igaf122.2996

**Published:** 2025-12-31

**Authors:** Courtney Gage, Elizabeth Rickenbach

**Affiliations:** Saint Anselm College, Concord, New Hampshire, United States; Saint Anselm College, Goffstown, New Hampshire, United States

## Abstract

Raising grandchildren has been linked to increased depression, stress, and worsened physical health. Research has shown that having social support may decrease stress and depression, but less is known about the impact of social support on physical health and chronic conditions among grandparent caregivers. The current study analyzed data from grandparent caregivers (n = 234) who participated in Wave 3 (2013) of the Midlife in the United States longitudinal study (Ryff., et al 2013). Correlational analysis showed that greater support from family correlated significantly with fewer chronic conditions (r=-.257, p = < 0.01), better physical health (r=-.275, p = < 0.01), and better mental/emotional health (r=-3.48, p = < 0.01). Greater support from friends predicted better physical health (r=-.163, p=.019). Independent samples t-tests were conducted to compare grandparents with high (4 or greater) versus low (3 or less) depressive symptoms. Support from family was higher among grandparents with fewer depressive symptoms (M = 3.53, SD =.56) as compared to grandparents with more depressive symptoms (M = 2.94, SD= .88) (t = 2.99, df = 21.87, p=.007). Similarly, support from friends was higher among grandparents with fewer depressive symptoms (M = 3.32, SD=.63) compared to grandparents with more depressive symptoms (M = 2.87, SD=.82) (t = 3.04, df = 22.74, p=.003). The findings show social support is important for grandparent caregivers’ physical and emotional health. This study demonstrates that increasing support groups and social assistance is important for grandparent caregivers.

